# Nutritional and Microbial Quality of Edible Insect Powder from Plant-Based Industrial By-Product and Fish Biowaste Diets

**DOI:** 10.3390/foods14071242

**Published:** 2025-04-02

**Authors:** Rafaela Andrade, Luisa Louro Martins, Miguel Pedro Mourato, Helena Lourenço, Ana Cristina Ramos, Cristina Roseiro, Nelson Pereira, Gonçalo J. Costa, Raphael Lucas, Nuno Alvarenga, João Reis, Ana Neves, Margarida Oliveira, Igor Dias, Marta Abreu

**Affiliations:** 1 Instituto Nacional de Investigação Agrária e Veterinária, Unidade de Tecnologia e Inovação, 2780-157 Oeiras, Portugal; andsrafaela@gmail.com (R.A.); cristina.ramos@iniav.pt (A.C.R.); cristina.roseiro@iniav.pt (C.R.); isa128286@isa.ulisboa.pt (N.P.); nuno.alvarenga@iniav.pt (N.A.); 2 LEAF—Linking Landscape, Environment, Agriculture and Food Research Center, Associated Laboratory TERRA, Instituto Superior de Agronomia, ULisboa, 1349-017 Lisboa, Portugal; luisalouro@isa.ulisboa.pt (L.L.M.); mmourato@isa.ulisboa.pt (M.P.M.); margarida.oliveira@esa.ipsantarem.pt.com (M.O.); 3 Instituto Português do Mar e da Atmosfera, I. P. (IPMA, I. P.), Avenida Alfredo Magalhães Ramalho 6, 1495-165 Algés, Portugal; helena@ipma.pt; 4 GeoBioTec—Geobiociências, Geoengenharias e Geotecnologias, NOVA School of Science and Technology, Universidade Nova de Lisboa, 2829-516 Caparica, Portugal; 5 The Cricket Farming Co., Quinta do Galinheiro, S. Pedro, 1001-904 Santarém, Portugal; goncalocosta@thecricketfarmingco.pt (G.J.C.); raphaellucas@thecricketfarmingco.pt (R.L.); 6 Escola Superior Agrária de Santarém, UI_IPS—Instituto Politécnico de Santarém, Quinta do Galinheiro, S. Pedro, 1001-904 Santarém, Portugal; joao.reis@esa.ipsantarem.pt (J.R.); ana.neves@esa.ipsantarem.pt (A.N.); igor.dias@esa.ipsantarem.pt (I.D.); 7 Centro de Estudos de Recursos Naturais Ambiente e Sociedade (CERNAS), Instituto Politécnico de Santarém, Quinta do Galinheiro, S. Pedro, 1001-904 Santarém, Portugal; 8 CIEQV—Life Quality Research Centre, Avenida Dr. Mário Soares n 110, 2040-413 Rio Maior, Portugal; 9 MED—Instituto Mediterrâneo para a Agricultura, Ambiente e Desenvolvimento & CHANGE–Global Change & Sustainability Institute, Universidade de Évora, Pólo da Mitra, Apartado 94, 7006-554 Évora, Portugal

**Keywords:** insect powder, house cricket, *Acheta domesticus*, alternative protein, sustainability protein, *Silurus glanis* by-products

## Abstract

Edible insect powder, particularly from the cricket *Acheta domesticus* L., is a promising sustainable alternative to traditional livestock-derived protein. Insects provide high protein content, fibre, and essential minerals, making them suitable for food applications. This study investigates the viability of alternative diets for rearing *A. domesticus*. Two experimental diets were tested: RI [50% horticultural by-products (HP) + 50% commercial diet (CD)] and RII (33% HP + 33% CD + 33% fish by-products). The results demonstrated that both diets were suitable for cricket rearing. Crickets reared on diets RI and RII produced, respectively, insect powders FI and FII, which were evaluated for their nutritional, bioactive, and microbiological attributes. Both powders exhibited high protein content (≈60%), all essential amino acids, higher mineral content than traditional protein sources, and met European Union food safety standards. Diet composition influenced powder characteristics: FI showed higher antioxidant activity and saturated fat content, while FII contained more protein, ash, minerals, and monounsaturated fatty acids. These findings underscore the potential of using industrial by-products to promote a circular economy in insect farming and suggest pathways for further research. However, since insects can bioaccumulate toxic elements, such as Hg, from diets, caution should be taken when considering fish by-products.

## 1. Introduction

According to the United Nations, current food production practices will not meet the global population’s needs, which is estimated to reach 9 billion by 2050 [1]. In this context, it is essential to invest in the development of food alternatives that are both nutritious and sustainable. Insects served historically as a staple food for many civilisations and still face resistance in numerous cultures. However, insect powder emerges as a promising food ingredient for human consumption, offering high nutritional value and more sustainable primary production compared to traditional livestock farming [1].

Insect powders generally contain significant amounts of high-quality protein (30–60%) [2], a balanced ratio of healthy fatty acids, and a beneficial amino acid profile. The specific composition will vary according to the insect species and the diets provided during their development [3,4], but insect proteins are generally digested well and readily absorbed by the human body, showing a digestibility comparable to other animal-based proteins [5].

Despite cultural resistance in some regions, the potential of insects to provide a sustainable nutrient source is undeniable. As awareness of their benefits grows, insects are poised to play a crucial role in addressing the global food supply challenges, ensuring a more resilient and sustainable food system for the future [1,6]. The European Union has already acknowledged four insects as novel foods: frozen, dried, and powdered forms of *Tenebrio molitor* larva (the yellow mealworm); frozen, dried, and powdered forms of *Locusta migratoria* (the migratory locust); frozen, dried, and powdered forms of *A. domesticus* (the house cricket); and *Alphitobius diaperinus* (the lesser mealworm). More insect species or derivatives are in the pipeline for approval [7].

In particular, powders made from house crickets (*A. domesticus*) have proven to be a suitable ingredient for the food industry, especially in bakery products, with up to 10% incorporation [8]. This species is frequently raised and used in insect powder production due to its favorable nutritional profile, which includes high-quality protein (with all essential amino acids), B vitamins, critical minerals (notably phosphorus, potassium, iron, and calcium), and beneficial fatty acids (predominantly mono- and polyunsaturated fats) [4]. Moreover, cricket powders have a neutral taste and color, facilitating their incorporation into various food products without negatively impacting flavor. This neutral profile helps increase consumer acceptance and reduce food neophobia regarding insect-based products.

The captive rearing of *A. domesticus* enables large-scale production of crickets and, in turn, of cricket powders; however, their quality depends significantly on rearing practices, with the insects’ diet playing a particularly critical role [4]. While some studies have explored the impact of different diets on the nutritional and functional quality of insect powder, gaps remain in understanding their effects on the product’s shelf life. The high costs associated with insect farming, particularly feed prices (such as chicken feed and soy), represent a significant barrier to large-scale production and the potential use of insects as food ingredients. Defining proper nutrition for insects is a critical research area, especially when considering agro-industrial by-products as alternative feed sources. By-products, such as those from minimally processed fruit and vegetable industries (including non-commercial parts like peels, stems, leaves, seeds, and stalks), possess high nutritional potential and are particularly rich in antioxidant compounds and fibre [9]. Also, fish by-products, mainly constituted of skin, bones, and cartilage, can be selected to enhance feed protein content. The use of these by-products contributes to their valorisation and promotes circular economy practices.

Safety concerns also arise, particularly regarding the potential presence of heavy metals in insect-based products, such as cadmium, lead, and mercury. While most studies suggest that these metals remain within safe limits for human consumption, concerns about bioaccumulation persist, highlighting the importance of diet selection for crickets, especially when testing alternative feeds.

This study aimed to examine the potential of alternative diets as partial replacements for commercial feed in the captive rearing of *A. domesticus* and assess their impact on the quality of the resulting insect powder. This study evaluated alternative diets as partial substitutes for commercial feeds in the captive rearing of *A. domesticus* crickets. Diet formulations were developed by incorporating industrial, fruit and vegetable, and fish by-products in varying proportions, selected based on their nutritional value and potential for circularity. The viability of these diets was assessed through a detailed analysis of the compositional and microbial attributes of the resulting insect powders.

## 2. Materials and Methods

### 2.1. Cricket Breeding Practices and Cricket Powder Production Methods

The cricket breeding and powder production were conducted on an industrial scale at The Cricket Farming Co. (Santarém, Portugal), following a standardised process. Initially, the eggs were carefully collected from the oviposition boxes and incubated under controlled conditions (temperature of 30 °C ± 2 °C and relative moisture content of approximately 60% ± 10%) until the nymphs hatched, which occurred within 8 to 10 days. The nymphs were then transferred to 800 mm × 600 mm × 400 mm plastic containers equipped with cardboard egg crates as structural support, feeders, and waterers, with a population density of 30 to 40 g of newly hatched nymphs with <24 h (about 45 k–60 k crickets) per container. They were fed daily with the corresponding diet and water ad libitum.

After approximately 50 days, the crickets reached maturity and entered the reproductive phase, during which the females laid eggs in oviposition boxes over 10 days, being replaced daily with fresh substrate. The rearing environment was continuously monitored to prevent contamination, with flypaper strips used to catch unwanted insects. After 60 days, the crickets were ready for harvest and processing. They were inactivated at 3 °C, followed by rigorous cleaning via water immersion, thermal blanching (80 °C for 6 min), and draining. The crickets were then frozen and stored at −18 °C.

For cricket powder preparation, the frozen crickets were thawed, pre-ground using a cutter CC-34 (Hällde, Stockholm, Sweden), dried in an industrial microwave MAX-30B (MAX Industrial Microwave, Yantai, China) at <100 °C for 15 min, and finally ground (200–300 μm) using a mill HC-400 (Cgoldenwall, Hangzhou, China). The resulting powder was vacuum-packed (≈1 kg), sealed until used, and stored at −80° C.

#### 2.1.1. Feed Formulation

Two distinct feed formulations were developed, using fruit and vegetable by-products (brassica leaves, tomato, and carrot tops), fish by-products (in the form of fishmeal derived from catfish (*Silurus glanis*) and other marine fish), and commercial chick feed A104. The proportions of the ingredients were as follows:

Diet I (RI): 50% fruit and vegetable by-products and 50% A104 feed;

Diet II (RII): 33% fruit and vegetable by-products, 33% fish by-products, and 33% A104 feed.

#### 2.1.2. Diets and Cricket Powders Assessment

The diets (RI and RII) and the corresponding cricket powders (FI and FII) were stored at −80 °C until analysis. The diet’s analytical protocol included proximate analysis, crude fibre, minerals, ash, and moisture content. For the cricket powders, the protocol covered proximate analysis, crude fibre, minerals, ash, moisture content, water activity (a_w_), total phenolic compounds (TPC), antioxidant activity (DPPH and FRAP methods), amino acid profile, fatty acid profile, and microbiological counts (Log CFU/g) for microorganisms at 30 °C and molds and yeasts.

### 2.2. Analytical Procedures

#### 2.2.1. Moisture Content, a_w_, Proximate Composition, and Caloric Value

Moisture content was measured according to the official AOAC method 925.10 [10] standard by drying the samples at 105 °C using an oven D-6450 (Heraeus, Hanau, Germany). Water activity (a_w_) was determined using a Hygrolab device (Rotronic, Bassersdorf, Switzerland). Ash content was quantified using a Thermolyne 48000 Muffle Furnace (Thermo Fisher Scientific, Waltham, MA, USA), based on the official AOAC method 923.03 [11]. Protein content was analysed using an NDA 702 Dumas Nitrogen Analyzer (VELP Scientific, Usmate Velate, Italy), equipped with a thermal conductivity detector (TCD), following the Dumas combustion method. The total nitrogen content was multiplied by a conversion factor of 6.25 to estimate the protein content.

Fat content was analysed following the method described by [12], with minor modifications. Two grams of each sample were extracted with 150 mL of n-hexane (Honeywell, Charlotte, NC, USA) using a Soxhlet apparatus DET-GRAS N 6p (J.P. Selecta, Barcelona, Spain) for 3 h. The solvent was then evaporated using an R-114 rotary evaporator (Büchi, Flawil, Switzerland), and the residue was dried overnight in an oven at 60 °C. After drying, the fat flask was cooled to room temperature and weighed. The fat content was determined by subtracting the tare weight from the final weight, dividing this value by the original sample mass, and expressing the result as a percentage. Carbohydrate content was calculated by difference using Equation (1).

% Carbohydrate = 100 − (% Moisture + % Protein + % Fat + % Ash)(1)

The total caloric value was calculated by summing the protein, lipid, and carbohydrate contents, applying the conversion factors outlined in Annex XIV of Regulation (EU) No. 1169/2011 [13].

#### 2.2.2. Crude Fibre

Crude fibre content was determined using the Weende method [14], with adaptations under the Association of Official Analytical Chemists method [15]. A 0.5 g portion of the dried sample was weighed and placed in a crucible fitted with a porous plate. Using the Fibertec apparatus (FOSS, Hillerød, Denmark), 150 mL of 1.25% sulfuric acid was added, and the sample was boiled for 30 min. After boiling, the sample was washed with hot distilled water to remove residual acid. Subsequently, 150 mL of 0.2 mol/L potassium hydroxide solution was added, and the sample was boiled again for 30 min using the Fibertec system.

After this step, the sample was vacuum-washed with acetone using a Kitasato flask. It was then dried in an oven at 103 °C and calcinated in a muffle furnace at 550 °C. The crude fibre content was calculated by the difference between the weight of the crucible with ash and the tare weight of the crucible, representing the crude fibre content. The results were expressed in grams per dry weight.

#### 2.2.3. Bioactive Composition

Extract Preparation: Extracts for the determination of total phenolic content (TPC) and antioxidant activity (AOx) were prepared from a 1:2.25 (*m*:*v*) mixture of the sample with methanol (100%; Honeywell, Charlotte, NC, USA). The mixture was homogenised at 20,000 rpm for 1 min using a Polytron Ultra-Turrax T 25 basic (IKA-Werke, Staufen, Germany) and then incubated in a 2200 Ultrasonic Cleaner (Branson Ultrasonics, Danbury, CT, USA) for 20 min. The samples were centrifuged at 7000 rpm for 20 min at 4 °C using a Sorvall RC5C with an SS34 rotor (Sorvall Instruments, Du Pont, Wilmington, DE, USA). The resulting supernatant was collected and stored at −20 °C until analysis.

Total Phenolic Content (TPC): The total phenolic content was determined with slight modifications to the procedure described by [16]. In test tubes, 2400 µL of distilled water, 150 µL of the extract, and 150 µL of 0.25 M Folin–Ciocalteu reagent (Sigma-Aldrich, St. Louis, MO, USA) were mixed and stirred. Then, 300 µL of 1 M sodium carbonate (Merck Millipore, Burlington, VT, USA) was added. The samples were allowed to react in the dark at room temperature for 2 h. After this incubation period, absorbance was measured at 725 nm using a UV/Vis spectrophotometer (Jas.co V-530, Tokyo, Japan). TPC results were expressed as milligrams of gallic acid equivalents per 100 g of DW (mg GAE/100 g DW).

Antioxidant Activity (AOx)—DPPH Method: The antioxidant activity was determined using the DPPH method based on the procedures described by [17,18], with slight modifications. A mixture of 150 µL of the extract and 2850 µL of DPPH solution (0.6 mol/L) (TCI Chemicals, Zwijndrecht, Belgium) was prepared. The samples were incubated at room temperature in the dark for 2 h. Absorbance was measured at 515 nm using a UV/Vis spectrophotometer (Jas.co V-530, Japan). Results were expressed as micromoles of Trolox equivalents per 100 g of DW (µmol TE/100 g DW).

FRAP Method: With some modifications, the FRAP method’s antioxidant activity was determined based on the protocol described by [19]. The working FRAP reagent solution was prepared by mixing 0.3 M sodium acetate buffer (pH 3.6) (Sigma-Aldrich, St. Louis, MO, USA), 10 mM TPTZ solution (Alfa Aesar, Haverhill, MA, USA), and 20 mM ferric chloride solution (Sigma-Aldrich, St. Louis, MO, USA) in a 10:1:1 ratio. A 0.2 mL aliquot of the extract was mixed with 1.8 mL of the FRAP reagent solution and incubated at room temperature for 5 min. Absorbance was measured at 593 nm using a UV/Vis spectrophotometer (Jas.co V-530, Japan). Results were expressed as millimoles of ferrous sulfate heptahydrate equivalents per 100 g of DW (mmol FeSO_4_·7H_2_O/100 g DW).

#### 2.2.4. Mineral Content

The mineral content of the samples was determined using inductively coupled plasma optical emission spectrometry (ICP-OES). Initially, the samples were dried in an oven at 80 °C for seven days. Approximately 0.3 g of each sample was weighed using an analytical balance (±0.001 g). For the acid digestion process, 8 mL of nitric acid (HNO_3_) and 2 mL of hydrochloric acid (HCl) were added to each sample. A blank test and a reference material (NIST Standard Reference Material 1570a, dried spinach leaves) were also prepared. After adding the acid solutions, the samples were subjected to digestion using a DigiPREP MS heating block (SCP Science, Baie-D’Urfe, QC, Canada) at 95 °C for two hours. After the two-hour digestion period, the samples were allowed to cool in a ventilated chamber until they reached room temperature. The cooled samples were then diluted to a final volume of 25 mL with ultra-pure water in volumetric flasks. The solutions were filtered through 90 mm diameter filter papers (Filter-Lab ref. 1242, Filtros Anoia, S.A., Barcelona, Spain). ICP-OES readings were conducted using an iCAP 7200 duo ICP-OES spectrometer (Thermo Fisher Scientific, Waltham, MA, USA) equipped with an autosampler.

Total mercury (Hg) content was determined using atomic absorption spectrometry with an automatic mercury analyser (LECO apparatus AMA 254, St. Joseph, MI, USA), following the EPA standard method 7473 [20]. For validation of the analytical method accuracy, dogfish liver-certified reference material for trace metals and other constituents (DOLT5–National Research Council of Canada, Ottawa, ON, Canada) was tested under the same conditions as the samples. The results obtained in this study were consistent with the certified values.

#### 2.2.5. Amino Acid Quantification

The amino acid profile was analysed using the HPLC method. A total of 0.5 g of each sample was weighed and placed in sealed Pyrex tubes containing 10 mL of 6 M hydrochloric acid (HCl). The samples were homogenised and then heated in an RVT 360 vacuum oven (Heraeus, Hanau, Germany) at 110 °C for 20 h. After cooling, the hydrolysate was filtered using 110 mm filter paper (Filter-Lab ref. 1238, Filtros Anoia, S.A., Barcelona, Spain), and the resulting filtrate was diluted to 100 mL with distilled water. For tryptophan quantification, basic hydrolysis was performed by adding 10 mL of 4 M sodium hydroxide (NaOH) to 0.1 g of sample. After heating at 110 °C for 20 h, the hydrolysate was filtered and diluted to 100 mL with distilled water. The solution was then neutralised to pH 7 using HCl and a pH meter (Metrohm, Herisau, Switzerland). Amino acid derivatisation was achieved by mixing 200 µL of the diluted hydrolysate (1:10,000) with 800 µL of o-phthalaldehyde (OPA) solution, followed by injection into the Alliance 2695 with a 2475 fluorescence detector HPLC system (Waters, Milford, CT, USA). Chromatographic separation was performed using a Spherisorb ODS2 column (4.6 mm × 250 mm, 5 μm) (Waters, Milford, CT, USA) at room temperature with a mobile phase consisting of solvent A, 0.1 M sodium acetate–methanol–tetrahydrofuran (905:90:5), and solvent B, methanol, employing gradient elution at a flow rate of 1 mL/min. Amino acids were detected by fluorescence with excitation at 340 nm and emission at 455 nm. Calibration curves were prepared for quantification, and results were expressed as g/100 g DW for each amino acid.

#### 2.2.6. Fatty Acids Profile

The samples’ fatty acid composition was analysed following the ISO 5508:1990 [21], using gas chromatography of fatty acid methyl esters (FAMEs). The fat samples underwent transesterification to convert triglyceride fatty acids into FAMEs, following the ISO 5509:2000 [22]. Analyses were performed using a Trace GC 2000 gas chromatograph (ThermoQuest, Milan, Italy), equipped with a split injector and a flame ionisation detector, utilising a DB-23 capillary column (60 m × 0.25 mm × 0.25 μm) (J&W Scientific, Agilent Technologies, St. Clara, CA, USA). Fatty acid identification was conducted by comparing retention times with reference standards of FAMEs. For quantitative analysis, the concentration of each fatty acid was expressed as a percentage, calculated as the ratio of the area of the peak corresponding to the fatty acid of interest to the total area of all peaks in the chromatogram.

#### 2.2.7. Fatty Acids Nutritional Indices

The health quality of *A. domesticus* lipids was evaluated by calculating the relationship between unsaturated and saturated fatty acids. The index of thrombogenicity (IT) is defined by the ratio of pro-thrombogenic (saturated) to anti-thrombogenic (unsaturated) fatty acids [23] and is calculated using Equation (2).

(2) $$IT=\frac{\left(C14:0+C16:0+C18:0\right)}{\left(0.5\times MUFA\right)+\left(0.5\times \sum n6PUFA\right)+\left(3\times \sum n3PUFA\right)+\frac{\sum n3PUFA}{\sum n6PUFA}}$$

The index of atherogenicity (IA) allows the evaluation of the lipid content in question and is the ratio of pro-atherogenic lipids (saturated) to anti-atherogenic (unsaturated) fatty acids. The formula used is based on studies by [23], with modifications. On the one hand, C12:0 was absent in said study, so it was not included in the formula. However, it is being considered for the calculation since it is on cricket’s lipidic content. On the other hand, C18:0 is not considered pro-atherogenic, as mentioned in the study by [24]. The IA is calculated using Equation (3).

(3) $$IA=\frac{C12:0+\left(4\times C14:0\right)+C16:0}{MUFA+\sum n3PUFA+\sum n6PUFA}$$

The potential effect on cardiovascular disease can be evaluated by the ratio between hypocholesterolemic and hypercholesterolemic fatty acids (H/H). The H/H ratio can be calculated using the following equation described by [23] (Equation (4)):

(4) $$H/H=\frac{C18:1\left(9\right)+C18:2\left(9,12\right)+C20:4\omega 6+C18:3\left(9,12,15\right)+C20:5+C22:5}{C14:0+C16:0}$$

Lastly, although omega-3 and omega-6 fatty acids are beneficial to human health, their imbalance can lead to health problems. As such, their ratio (ω6/ω3) is calculated to evaluate omega fatty acids balance (Equation (5)), written as follows:

(5) $$\frac{\omega 6}{\omega 3}=\frac{\sum n6PUFA}{\sum n3PUFA}$$

### 2.3. Microbial Analysis

The microorganism at 30 °C and the mold and yeast counts were performed according to ISO 4833-1:2013 [25] and ISO 21527-1:2008 [26] standards, respectively. Initially, the samples were suspended in tryptone salt (1:10) and homogenised in a Stomacher 400 (Lab Blender, Seward, UK) using ionised filter bags, model BBAG-03 190 mm × 300 mm − 400 mL (Interscience, Paris, France). Subsequently, successive decimal dilutions were made from 10^−1^ to 10^−7^.

For the microorganisms at 30 °C, the pour plate technique was used with Plate Count Agar (PCA) as the culture medium, and the plates were inverted and incubated in an 854 incubator (Memmert, Schwabach, Germany) at 30 °C ± 1 °C for 72 ± 3 h. For mold and yeast counts, the spread plate technique was applied with Dichloran Rose Bengal Chloramphenicol (DRBC) as the culture medium, and the plates were incubated in an 854 incubator (Memmert, Schwabach, Germany) at 22 °C ± 1 °C for 5 days.

### 2.4. Statistical Analysis

Data were analysed using factorial ANOVA in Statistica™ v8.0 [27], with statistically significant differences identified by Tukey’s HSD test at a significance level of *p* < 0.05. Pearson correlation coefficients assessed relationships among selected responses. Principal component analysis (PCA) was also performed after mean-centering and standardising all variables to unit variance (correlation matrix). Principal components were extracted by calculating eigenvalues and eigenvectors from the dataset’s correlation matrix [28], and sample scores for each PCA component were computed as linear combinations of the variables. Factor loadings represented each variable’s contribution to the PCA, highlighting their influence on the components. A two-dimensional plot was generated for the primary components, capturing over 70% of the original data variability, deemed sufficient for qualitative modeling [29]. Hierarchical cluster analysis followed, involving data standardisation, dissimilarity calculation (Euclidean distance), and application of Ward’s method as the clustering technique.

## 3. Results and Discussion

### 3.1. Insect Diet Formulations

#### 3.1.1. Proximal Analysis, Crude Fibre, and Caloric Value

Table 1 presents the mean values (±SD) of the proximal analysis, crude fibre, and caloric value of the RI and RII diets. Significant differences (*p* < 0.05) were observed in all parameters except protein content. The higher moisture content in RII (72.5%) compared to RI (53.3%) can be attributed to the formation of ice crystals during the freezing process of fishmeal, causing the release of water during thawing.

The protein content of diets RI and RII (9%) is below the recommended range of 20% to 30% for cricket rearing [30,31]. Oonincx et al. [32] found that diets with less than 20% protein can support cricket survival and development. This finding is corroborated in the present study, in which the insects completed their life cycle in 60 days. Insect protein utilisation is also influenced by additional factors, including the amino acid profile [33]. In this regard, it is recommended that diets comprise a variety of protein sources, as this facilitates a balanced and diverse amino acid profile.

The disparity in lipid content between RI (3.7%) and RII (26.1%) can be attributed to the incorporation of fish residues into RII. It is important to note that the fish biowaste used does not correspond to muscle tissue but to parts typically richer in lipids, such as the skin and viscera. Although the fat content in muscle is generally below 10%, these discarded parts naturally possess a much higher fat concentration, which could explain the obtained result. Conversely, RI complies with the recommended range of 3% to 5% fat [30].

The carbohydrate content of the RII diet was lower (52.7%) than the RI diet (81.4%). Patton, R.L. [30] posited that diets comprising 32% and 47% carbohydrates are suitable for rearing domestic crickets. The commercial chick feed A104, employed in formulating the RI and RII diets, consists of grains rich in carbohydrates, with the three principal ingredients being corn, soybean meal, and wheat. The higher proportion of chick feed in RI (50%) compared to RII (33%) may be the reason for the elevated carbohydrate content in the former.

On the other hand, the fibre content was higher in RII (11.7%) than in RI (6.2%). In the study by [34], two commercial feeds designed explicitly for large-scale cricket farming were tested, with fibre contents ranging between 5.3% and 5.9% on a DW basis. The fibre contents in RI were found to be similar to the values mentioned above, while those in RII were observed to be higher. By-products from the horticultural industry, which include various plant parts such as brassica stalks and leafy vegetables, are known to contain high levels of fibre. The different fibre compositions of the two diets result in substantial variability, which renders a direct comparison of their component proportions challenging, particularly given the differing incorporation levels of this component, with 50% in RI and 33% in RII.

Regarding caloric value, the RII diet exhibited a higher value (482.3 kcal) than the RI diet (394.4 kcal), representing an increase of approximately 20%. This increase can be attributed to the higher lipid content of RII, as lipids have a substantial impact on caloric calculations due to their higher energy contribution. The caloric conversion factor for lipids is 9 kcal per gram, whereas for carbohydrates, it is 4 kcal per gram. Therefore, the elevated lipid content in RII considerably impacted the total energy value, rendering it more calorically dense than RI.

#### 3.1.2. Mineral Content

The mineral composition of RI and RII diets (Table 2) demonstrates that the levels of the predominant macronutrients (Ca, P, S, Na, and Mg) as well as Fe are significantly higher (*p* < 0.05) in diet RII compared to diet RI. The exception is potassium, which demonstrated similar values in both diets. Furthermore, the elevated ash content observed in diet RII provides additional evidence to support this relationship between the mineral contents of the diets. By contrast, the copper and manganese micronutrients display markedly reduced levels in diet RII.

The disparity in mineral composition between the two diets can be ascribed to the disparate formulations and ingredients utilised in their formulation, RI and RII, respectively. The mercury concentration (Hg) in RII (0.015 ppm), although higher than in RI, remains within the regulatory limits (0.1 ppm). However, the notable enhancement in the levels of essential minerals, including sodium (Na), calcium (Ca), magnesium (Mg), phosphorus (P), sulfur (S), and iron (Fe), in RII in comparison to RI could confer nutritional advantages. These minerals are essential for various physiological processes, making RII potentially more advantageous from a nutritional standpoint despite its higher mercury content within the regulatory limits.

Oonincx et al. [32] emphasised the importance of evaluating the phosphorus content within insect diets, given that elevated levels are thought to positively influence the life cycle characteristics of insects, including that of the house cricket. Visanuvimol et al. [35] reported that expected phosphorus levels should range between 0.2% and 1% in *A. domesticus* diets. As illustrated in Table 2, the phosphorus concentration in diet RI equates to 7000 ppm (approximately 0.7%), falling within the recommended range. Diet RII, however, contained 22,948 ppm (approximately 2%), which exceeds the recommended range. The elevated phosphorus levels can be attributed to incorporating 33% fish in the RII. Nevertheless, this increased phosphorus content did not seem to have a deleterious effect on cricket rearing.

Directive 2002/32/EC establishes maximum limits for undesirable substances in animal feed, applicable across species. Regarding toxic elements in diets, including those suitable for insects, the permitted maximum levels are as follows: Pb—10 mg/kg, Cd—1 mg/kg, Hg—0.1 mg/kg, and As—2 mg/kg [36]. The levels of toxic elements in diets RI and RII (Table 2) were found to be below 1 mg/kg DW for cadmium and lead and below 1.5 mg/kg DW for arsenic and nickel, indicating that the tested diets comply with the established limits for this type of contamination.

The mercury (Hg) detected in diet RI was found at levels below the limit of quantification (LQ ≤ 0.01 mg/kg), likely without toxicological relevance. Conversely, the Hg levels obtained in diet RII were quantifiable but were within acceptable limits as stipulated in Directive 2002/32/EC [36]. While the concentrations observed in the RII diet remain below the regulatory limits, these potentially toxic elements warrant further investigation and may be attributed to incorporating fish by-products, constituting 33% of the diet’s composition.

Fish are known to bioaccumulate Hg in their tissues, which may explain the levels detected in the RII diet’s analysis. This fact underscores the importance of closely monitoring the presence of toxic elements in marine-derived ingredients, mainly when used in formulations intended for animal feed, to ensure compliance with food safety standards.

### 3.2. Insect Powder Composition

#### 3.2.1. Proximal Analysis, a_w_, Crude Fibre, and Caloric Value

The proximal analysis and caloric value of the insect powders from *A. domesticus* (FI and FII) are shown in Table 3.

The moisture content and water activity (a_w_) values of samples FI and FII are found to differ insignificantly (*p* > 0.05), remaining below 5% and 0.4%, respectively. Udomsil et al. [37] reported higher moisture values (6.3%) in insect flours from the same species. The reduced moisture and aw values are particularly effective in preventing the growth of common bacteria, yeasts, and molds, which can otherwise cause food to spoil [38]. Therefore, low water content is important in preserving insect powder, ensuring its stability at room temperature.

The proximate composition analysis of the insect powders (Table 3) revealed that protein is the predominant component, accounting for over 55% of the total composition, followed by carbohydrates, lipids, and ash in decreasing proportions. Significant differences (*p* < 0.05) were identified in the protein content and ash between the samples, with sample FII exhibiting the highest values. However, both samples exhibited similar percentages of carbohydrates, lipids, and caloric values (kcal). The proportion of carbohydrates in the samples (approximately 19% in FI and 17% in FII) closely aligns with the fibre content (approximately 15% in FI and 16% in FII), indicating that the carbohydrate fraction is predominantly composed of fibre.

In similar studies, reported protein contents ranged from 62.4% to 71.1% [3,39], which are higher than the values observed in samples FI (57.4%) and FII (60.8%). Both authors reported values between 9.8% and 22.8% regarding lipid content, aligning with our findings. The ash content reported in the mentioned studies varied from 3.6% to 9.1%, comparable to 4.4% in FI samples and 4.8% in the FII samples. For fibre content, the values found in the literature ranged from 10.2% to 22.1%, with our results of 14.8% for FI and 16.4% for FII comfortably situated within this range. Concerning caloric value, the cited studies reported approximately 455 kcal/100 g, similar to the calculated values for FI (433.2 kcal/100 g) and FII (430.6 kcal/100 g).

#### 3.2.2. Amino Acid Profile

The amino acid profile (Table 4) presents the identified and quantified essential amino acids (EAAs) and non-essential amino acids (NEAAs), along with the total sum of essential amino acids (∑EAAs) and non-essential amino acids (∑NEAAs), for *A. domesticus* insect powders (FI and FII).

As shown in Table 4, both insect powders contain all nine EAAs, indicating them as protein sources comparable to traditional sources, such as egg, chicken, pork, and beef [37].

The EAA content was similar (*p* > 0.05) between samples, except for valine and isoleucine, which both showed significantly higher levels in sample FI (*p* < 0.05). The quantified EAA levels (Table 4) are consistent with those reported in the study of [37] on *A. domesticus* cricket powder. The study also observed that tryptophan exhibited the lowest amino acid concentration relative to the other amino acids, much like what is observed for FI and FII.

The identified NEAAs included aspartic acid, glutamic acid, serine, glycine, arginine, alanine, and tyrosine. The NEAA content was comparable between the FI and FII insect powder samples (*p* > 0.05), except for serine, glycine, and alanine, which showed significantly higher concentrations in the FI samples (*p* < 0.05). The significant differences in serine, glycine, and alanine in the profiles of samples FI and FII can be attributed to the compositional differences in the cricket diets, namely the presence of fish biowaste in RII. Udomsil et al. [37] also reported the presence of NEAAs in cricket powders. However, discrepancies were noted; our study did not identify proline and cysteine in insect powders due to the derivatisation method used.

The most abundant essential amino acids (>3 g/100 g) in both powders were valine, leucine, phenylalanine, and lysine. In FI, isoleucine also exhibited content within this range (Table 4).

The lysine and threonine contents identified in both powders (Table 4) were similar to those quantified by [37] in *A. domesticus* powder. This finding is important, making this protein source a possible complement for cereal-based diets, which are generally low in these amino acids [40].

Among the NEAAs, the most abundant were glutamic acid, arginine, and tyrosine (>3 g/100 g) for both powders. Alanine and serine also showed elevated levels in the FI powder (Table 4). Previous studies involving various insect species [3,41] have shown similar NEAA profiles. Among them, the presence of glutamic acid is of great importance since the high presence of this amino acid is typically associated with the umami flavor characteristic of insect consumption.

While glutamic acid is important for food flavor, the detected high levels of arginine in FI and FII powders (>3 g/100 g) are significant for the human diet, as the synthesis of this amino acid in humans is generally insufficient, particularly during growth and childhood development [41].

Udomsil et al. [37] reported that crickets generally contain low levels of methionine, tryptophan, and cysteine, a pattern also observed in the FI and FII powders. The same authors noted that low levels of lysine and tryptophan also characterise most edible insects. However, significant levels of lysine were evaluated in the FI and FII powders.

The total amino acids (sum of EAAs and NEAAs) were significantly higher (*p* < 0.05) in the FI powder compared to the FII powder, which may be partially attributed to the differences in the insects’ diets (RI and RII) [41].

The protein quality of edible insects in the human diet can be assessed by their amino acid (AA) content, with particular emphasis on essential amino acids (EAAs), whose presence is a crucial indicator of protein quality [42], underscoring their importance in human nutrition. Considering the estimates from the World Health Organization (WHO) cited by [43] for the daily essential amino acid requirements per kg of body weight in healthy adults and considering the consumption of 100 g of the final product (FI or FII) for a 70 kg adult, the amino acid profiles of the FI and FII powders suggest their potential as dietary supplements, supplying satisfactory amounts of essential amino acids for human health.

#### 3.2.3. Fatty Acids Profile

The fatty acid profile of *A. domesticus* insect powders (FI and FII) is presented in Table 5. It shows the different fatty acids identified and quantified (mean values ± SD) for those exceeding 5% of the total fat content.

In both samples, the dominant fatty acids, in descending order, were linoleic acid [C18:2 (9, 12)], palmitic acid (C16), oleic acid [C18:1 (9)], and stearic acid (C18). Except for oleic acid, which showed similar values (around 24 g/100 g of fat) in both samples, the levels of the other fatty acids were higher in the FI samples compared to the FII samples.

The fatty acid profile, particularly regarding oleic and linoleic acid, suggests that *A. domesticus* insect powder may serve as a valuable nutritional source, providing essential fatty acids that the human body cannot synthesise and are essential in the human diet.

Linoleic acid is the predominant fatty acid in both samples (Table 5). This polyunsaturated fatty acid (PUFA), which belongs to the omega-6 family of fatty acids, plays several physiological roles that are essential for human health, including normal growth and development, as well as the maintenance of skin and hair health [44,45].

Furthermore, the ratio of these fatty acids is of significance, with particular attention paid to the ratio of monounsaturated to polyunsaturated fatty acids (MUFA/PUFA), along with the presence of saturated fatty acids (SFA), as illustrated in Table 6. The optimal proportion of saturated, monounsaturated, and polyunsaturated fats in a diet is 1:1:1. An even distribution of nutrients is crucial for health. It reduces the risk of cardiovascular diseases, improves the inflammatory response, and promotes overall health [46].

Except for the PUFA, which exhibited comparable proportions across the samples, the MUFA ratio was higher (*p* < 0.05) in sample FII, whereas the SFA ratio was higher (*p* < 0.05) in sample FI. As seen in Table 6, both insect powders display a ratio of SFA, MUFA, and PUFA close to the ideal ratio of 1:1:1. Sample FII, in particular, is notable for having a lower proportion of saturated fat.

The results of previous studies corroborate these findings, indicating that the fatty acids present in this cricket species conform to the specified proportions, thereby establishing them as a healthy source of lipids [46,47].

The exclusive presence of eicosapentaenoic acid (C20:5 EPA) and docosahexaenoic acid (C22:6 DHA) in the FII samples (Table 5), although in low concentrations, may be attributed to dietary variations in the insects’ diet. These fatty acids are predominantly present in seafood and derived products, and only the RII diet contained fish by-products. Similar outcomes were documented by [3], who identified omega-3 fatty acids, specifically eicosapentaenoic acid (EPA) and docosahexaenoic acid (DHA), in *Hermetia illucens* larvae that had been fed fish residues. These results provide further evidence of the influence of insect diet on the quality of insect powder.

#### 3.2.4. Fatty Acids Nutritional Indices

The relationship between unsaturated and saturated fatty acids allows for assessing a foodstuff’s dietary quality, thrombogenicity, atherogenicity, and effect on cardiovascular diseases. As mentioned, said relationships were estimated by calculating IT, IA, H/H, and the ratio ω6/ω3 for FI and FII’s fatty acid content. IT, IA, and H/H are helpful indices for directly comparing food fatty acid contents [48].

Atherogenicity is the formation or accumulation of abnormal fatty deposits on the innermost layers of arteries. Thrombogenesis is the formation of a thrombus within a blood vessel. Some compounds, including certain fatty acids, can inhibit or prevent atherogenicity and/or thrombogenesis [49]. For IT and IA, lower values indicate a product is better for human health since there will be more UFA than SFA, which has a preventative effect. For H/H, a higher value is better since it shows a higher content of hypocholesterolemic fatty acids [49,50,51].

As seen in Table 7, although the values for IA and H/H have an apparent positive change between the cricket powders, that difference has no statistical significance. This means that the change in the feed may improve these indices, potentially turning the cricket powder more beneficial for human health by not contributing significantly to the incidence of pathogenic phenomena, such as atheroma and/or thrombus formation [50]. However, this effect should be further studied to determine if using fish by-products can lead to significant differences, especially in higher concentrations. As for IT, there are significant differences, such as a decrease in value, indicating a healthier option in FII compared to FI.

Table 8 shows IA, IT, and H/H values from various foods, adapted from [52,53], and including present study powders. FI and FII present quite appealing values, showing below-average values of IA and a relatively good value of H/H and IT. Our powders have values like those of river fish and show better values than milk. It can also be noted that olive oil is a remarkable food in what relates to these indices.

The ω6/ω3 PUFA ratio shows a different behaviour. It still shows a positive outlook from FI to FII but with significant differences. Furthermore, the incorporation of fish by-products in the feed led to an increase in omega-3 PUFAs (1.92 ± 0.30 mg/kg to 4.00 ± 0.38 mg/kg) and a simultaneous decrease in omega-6 PUFAs (31.1 ± 0.95 mg/kg to 26.8 ± 2.78 mg/kg), decreasing the ratio to a value closer to a value considered ideal for human consumption of 4 [51] or 5 [50]. This change can be mainly attributed to the decrease in C18:2 (9, 12), the increase in C18:3 (9, 12, 15), and the appearance of fish-related FA C20:5 EPA, C22:6, and C20:4 ω3. Scientific evidence shows that high values of the ω6/ω3 PUFA ratio may lead to the increase of pathogenesis of many diseases, like cardiovascular disease [54]. As such, incorporating fish by-products into the feed led to healthier insect powder, demonstrating the possibility of obtaining a more customised cricket powder depending on the feed.

#### 3.2.5. Bioactive Composition

Table 9 presents the mean results (±SD) for antioxidant activity (AOx) and total phenolic content (TPC) in *A. domesticus* insect powders (FI and FII).

The FI sample exhibited significantly higher AOx, measured by the FRAP method (72.9 ± 1.9 mmol FeSO_4_·7H_2_O/100 g DW), compared to FII (43.0 ± 3.8 mmol FeSO_4_·7H_2_O/100 g DW), nearly double the value. Similarly, the DPPH method results showed that the FI sample (5055.0 ± 2.3 µmol TE/100 g DW) had significantly greater AOx than FII (4928.5 ± 21 µmol TE/100 g DW), with only a 3% discrepancy. In contrast, Pilco-Romero et al. [4] reported lower AOx values for *A. domesticus* cricket powder, ranging from 1700 to 2030 µmol TE/100 g.

Regarding TPC, the FI sample (212.0 ± 24.9 mg GAE/100 g DW) was significantly higher (*p* < 0.05) than the FII sample (120.9 ± 5.5 mg GAE/100 g DW), indicating an almost twofold difference. A strong positive correlation (r = 0.9; *p* < 0.05) between TPC and AOx suggests that the antioxidant compounds in the insect powders are predominantly phenolic.

Neveu et al. [55] identified various antioxidant compounds in domestic cricket powders, including phenolic compounds. Carlsen et al. [56] noted that the AOx of these insect powders is comparable to, and sometimes exceeds, those of other foodstuffs, such as beverages, spices, herbs, and dietary supplements. Nino et al. [57] detailed the profile of antioxidants and phenolic compounds in *A. domesticus*, highlighting the presence of hydroxybenzoic acids (e.g., gallic, syringic, and 2,4-dihydroxybenzoic acids) and hydroxycinnamic acids (e.g., chlorogenic, caffeic, *p*-coumaric, and ferulic acids). Cyclohexanecarboxylic acid (quinic acid) was also identified, along with four flavonoids: apigenin (flavone), daidzein (isoflavone), quercetin (flavonol), and naringenin (flavanone).

These phytochemicals, typically found in plant-based products, suggest domestic crickets can metabolise, absorb, and store phenolic compounds from their diet. The RI feed formulation, which contains 50% fruit and vegetable by-products, resulted in a double TPC powder compared to RII feed, which had about 33% fruit and vegetable by-products.

While further studies are required to validate this hypothesis, preliminary results are promising, indicating that insect powders derived from *A. domesticus* can be considered nutrient-rich foods—providing proteins and lipids—and natural sources of functional phytochemicals.

#### 3.2.6. Mineral Content

The mineral compositions of the two cricket powder samples are shown in Table 10.

The mineral composition of the two cricket powder samples is similar, with the mineral contents presented in the following descending order: potassium (31%), phosphorus (30%), sulfur (17%), sodium (12%), calcium (7%), and magnesium (4%). The quantities of other identified minerals, including chromium, copper, zinc, iron, manganese, and cadmium, were significantly lower (<0.02%).

The levels of predominant minerals are significantly higher (*p* < 0.05) in sample FII compared to FI, except for calcium and sodium, which exhibited similar values in both samples. The mineral composition of *A. domesticus* powders reported by [3,4] is consistent with that found in this study, highlighting potassium, phosphorus, sodium, calcium, and magnesium in the same order of predominance. However, sulfur was not identified in the analyses conducted by these authors. The reported ranges also align with those of this study.

The observed differences between samples FI and FII could be attributed, at least in part, to differences in diet (RI and RII), which displayed distinct mineral compositions, particularly in phosphorus, potassium, and calcium (Table 2).

Nevertheless, the relationship between the mineral composition of the diets and the resulting insect powders is not entirely straightforward. The RI feed exhibited reduced calcium and phosphorus levels (*p* < 0.05) compared to the RII diet. The resulting insect powders showed discrepancies in phosphorus content, with the FI insect powder demonstrating a lower level of this mineral relative to FII, but the calcium content was not significantly different.

Concerning identifying toxic elements in the FI and FII powders, the measured concentrations were below 1 mg/kg for cadmium and lead and below 1.5 mg/kg for arsenic and nickel. According to the specifications outlined in Regulation (EU) 2022/188, the maximum permissible limits for lead and cadmium in cricket powder are set at 0.05 mg/kg and 0.06 mg/kg, respectively [58]. However, since the method lacked the sensitivity required to detect lower concentrations of these elements, employing more precise analytical methods to quantify these minerals and accurately confirm compliance with regulatory limits would be necessary.

Regarding mercury, the average levels in the FI and FII powders showed significant differences (*p* < 0.05), with values of 0.02 ± 0.01 mg/kg DW and 0.228 ± 0.07 mg/kg DW, respectively (Table 10).

The EU Regulation 2022/188 for using *A. domesticus* powder as a novel food does not specify values for the presence of mercury (Hg) [58]. Therefore, for such products, the maximum permitted limit of 0.10 ppm, as stated in Regulation (EU) 2023/915 [59] for contaminants in foodstuffs, may apply, given that cricket powder falls under point 3.3.2 (dietary supplements) of this regulation. In this context, only the FI powder meets the regulatory requirements. In contrast, the FII powder exceeds the established limit, with a value of 0.228 mg/kg DW, which may raise concerns regarding food safety.

The difference in mercury levels between the insect powders can be explained by the distinct compositions of the diets provided to the crickets (RI and RII). The higher levels of mercury in the FII insect powder are associated with a higher concentration of Hg in the RII diet (Table 2), suggesting that bioaccumulation may have occurred in the insects fed this diet. This phenomenon is consistent with studies demonstrating the bioaccumulation of heavy metals in various insect species, particularly when exposed to contaminated diets or environments, especially in organisms situated at higher levels of the food chain.

FI powder results are similar to those of [60], who found 0.028 ppm mercury in cricket powders. These findings emphasise the necessity of comprehensive monitoring of heavy metals in insect-based products to ensure regulatory compliance and consumer safety. Given the potential for bioaccumulation, it is vital to undertake a comprehensive dietary source assessment of insects used in food products to minimise the risk of heavy metal contamination.

The mineral content of *A. domesticus* cricket powder makes it an essential source of nutrients for children and healthy adults, providing a significant portion of the recommended daily intake (DRI) for macrominerals, such as calcium, magnesium, phosphorus, potassium, and sodium [61]. A similar benefit is observed for essential trace minerals in the human diet, including chromium, copper, zinc, iron, and manganese. In this regard, *A. domesticus* cricket powder is an important mineral source, except for selenium and iodine, which were undetected. Furthermore, the macronutrients and micronutrients in domestic crickets exceed those typically found in chicken breast [62] and pork loin [63].

#### 3.2.7. Microbial Evaluation

The microbiological evaluation (Table 11) revealed similar results for the FI and FII samples (*p* > 0.05). The microorganisms at 30 °C counts ranged between 3.2 and 3.6 log10 CFU/g FP, while the mold and yeast counts ranged from 0.3 to 1 log10 CFU/g FP. These values comply with the limits established by Regulation (EU) 2022/188 [58] for *A. domesticus* cricket powder, which specifies ≤ 5 log10 CFU/g for mesophilic bacteria and ≤2 log10 CFU/g for molds and yeasts.

Compliance with the two microbiological criteria analysed, as established in the cricket powder regulation, highlights the product’s microbiological quality concerning these specific parameters. These results suggest that good hygiene practices were implemented during the rearing and processing stages, contributing to the safety of the final product for human consumption. However, it should be noted that a more comprehensive assessment of microbiological compliance would require analysing the remaining criteria stipulated by the regulation.

### 3.3. Relationship Between Cricket Diet and Insect Powder Quality

The relationships between the composition of the feeds (RI and RII) and the final powders (FI and FII) were explored using a principal component analysis (PCA) and a hierarchical cluster analysis. The data matrix incorporated all the variables commonly assessed in the sample types while solely considering the most pertinent minerals within the mineral profile. As a result, the data matrix included 13 quantitative variables (moisture, protein, lipids, carbohydrates, fibre, ash, caloric value, sodium, potassium, calcium, magnesium, phosphorus, and sulfur) along with 12 sample types, referred to as RI (n = 3), RII (n = 3), FI (n = 3), and FII (n = 3).

**Figure 1:**
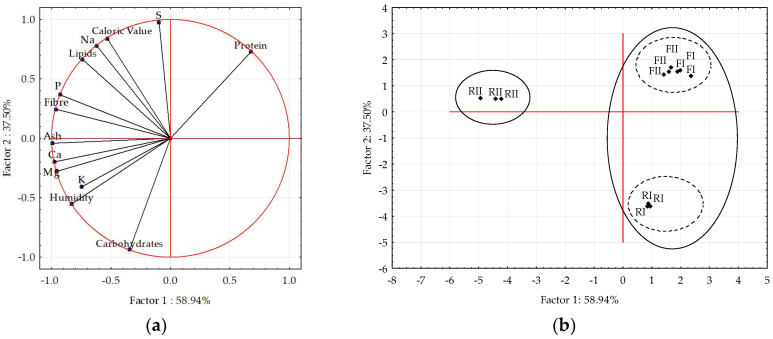
Principal component analysis (PCA) of feed samples (RI and RII) and the *A. domesticus* powders (FI and FII): (**a**) Variable vector plot; (**b**) Sample score plot. In the cluster analysis (“complete linkage”), the first separation occurs at a Euclidean distance of 5.25 (solid line), while the second separation occurs at a Euclidean distance of 4.25 (dashed line).

The PCA analysis revealed that the first two dimensions (principal components, PC1 and PC2) collectively accounted for 96.44% of the total data variability, which aligns with the threshold deemed adequate for a qualitative model [29]. A summary of the factor loadings for the first two principal components across all assessed variables is presented in Supplementary Material Table S1. Figure 1 presents the spatial projection graphs, which demonstrate the configuration of variable vectors (a) and the dispersion of samples (b) within the two-dimensional space defined by the first two principal components.

The first principal component (PC1) accounted for 58.94% of the data variability, while the second principal component (PC2) explained 37.50%. For PC1, the variables that contributed significantly include moisture, lipids, fibre, ash, and the minerals potassium (K), calcium (Ca), magnesium (Mg), and phosphorus (P), all of which exhibited negative loadings. Therefore, the gradient represented by PC1 can be interpreted accordingly. The samples were positioned along PC1 [see Figure 1a] by decreasing concentrations of moisture, lipids, fibre, ash, and the minerals K, Ca, Mg, and P from left to right.

The second principal component (PC2) was significantly influenced by protein, caloric value, sodium (Na), and sulfur (S), which had positive loadings, while carbohydrates exhibited a negative loading. Therefore, PC2 can be considered to represent a concentration gradient. The higher-level samples displayed increased protein, caloric value, sodium (Na), and sulfur (S), while the lower-level samples exhibited elevated carbohydrate concentrations.

Figure 1b illustrates the clustering of samples based on the principal components PC1 and PC2, as determined by hierarchical cluster analysis. At the initial clustering level (Euclidean distance of 5.25), two groups emerged: one comprising the RI, FI, and FII samples and the other containing the RII samples. The F and RI samples showed clear separation at the second level (Euclidean distance of 4.25).

Based on the comparative sample’s positioning, the RI feed (quadrant II) was characterised by its high carbohydrate content, lower protein levels, caloric value, and sodium and sulfur minerals. Conversely, the RII feed (quadrant IV) was noted for its higher moisture, lipid, fibre, ash, and mineral content, including calcium, magnesium, potassium, and phosphorus. In contrast, the cricket powder samples (FI and FII) in quadrant I were recognised for their high protein content.

Given the marked divergence in the sample’s nature, the separation between the feed samples (RI and RII) and the cricket powders was to be expected, given that the insect powders are dehydrated products with significantly higher protein content. Nevertheless, it is crucial to underscore that, despite their markedly disparate compositions, the feed samples (RI and RII) yielded flours with analogous compositions grouped in the same cluster. This finding demonstrates that both feeds were suitable for cricket rearing despite the pronounced differences in their composition.

## 4. Conclusions

This study confirms the viability of alternative diets formulated for rearing *A. domesticus*, demonstrating their substantial influence on the composition of the resulting cricket powder. Based on industrial by-products (RI and RII), both feeds successfully supported cricket growth. This approach aligns well with circular economy principles, offering a sustainable avenue for developing novel products in human nutrition. The two feeds exhibited distinct compositions: RI was richer in carbohydrates, while RII had higher caloric density, moisture, lipids, fibre, ash, and minerals. Remarkably, the cricket powder attained approximately 60% protein content despite the low-protein composition of the feeds (~9%), suggesting that protein levels in insect powders were pretty independent of dietary protein content. These finding challenge earlier research advocating for high-protein diets to achieve similar protein concentrations in cricket powder.

The cricket powder also exhibited high protein quality, a balanced amino acid profile—including all nine essential amino acids, and a robust mineral content, meeting the microbiological standards set by Regulation (EU) 2022/188 for *A. domesticus* powder [58]. The feed composition significantly influenced the properties of the cricket powders. The FI cricket powder (from feed RI) showed higher antioxidant activity and saturated fatty acids and amino acids concentrations. In contrast, the FII cricket powder (from feed RII) contained higher protein levels, ash, minerals, and monounsaturated fatty acids. Notably, although mercury (Hg) levels in feed RII were within regulatory limits, the FII cricket powder exceeded the permitted Hg concentrations due to bioaccumulation, raising a critical food safety concern.

## Figures and Tables

**Table 1:** Average values (±standard deviation, SD) of moisture content (% fresh weight, FW), proximal composition and crude fibre (% dry weight, DW), and caloric value (kcal) in diets RI and RII.

Parameters	RI	RII
Moisture content (%, FW)	53.3 ± 0.1 ^b^	72.5 ± 0.8 ^a^
Protein (%, DW)	8.9 ± 0.8 ^a^	9.1 ± 0.1 ^a^
Lipids (%, DW)	3.7 ± 0.1 ^b^	26.1 ± 0.3 ^a^
Carbohydrates (%, DW)	81.4 ± 0.9 ^a^	52.7 ± 0.6 ^b^
Ash (%, DW)	6.0 ± 0.2 ^b^	12.1 ± 0.3 ^a^
Crude fibre (%, DW)	6.2 ± 1.0 ^b^	11.7 ± 0.4 ^a^
Caloric value (kcal)	394.4 ± 1.3 ^b^	482.3 ± 0.5 ^a^

Note: Different letters in a given row indicate significant differences at *p* < 0.05 (Tukey test).

**Table 2:** Mean (±SD) mineral content (mg/kg DW) in diets RI and RII.

Minerals (mg/kg DW)	RI	RII
Sodium—Na	1880.8 ± 38.7 ^b^	5372.7 ± 293.2 ^a^
Potassium—K	12,012.7 ± 217.3 ^a^	12,604.4 ± 621.2 ^a^
Calcium—Ca	9590.9 ± 1063.5 ^b^	23,995.2 ± 2481.4 ^a^
Magnesium—Mg	1831.5 ± 52.1 ^b^	2655.3 ± 205.4 ^a^
Phosphorus—P	7000.6 ± 100.8 ^b^	22,978.8 ± 1594.6 ^a^
Sulfur—S	2729.6 ± 56.6 ^b^	5434.6 ± 331.8 ^a^
Chromium—Cr	4.5 ± 0.1 ^a^	4.1 ± 0.3 ^a^
Copper—Cu	14.3 ± 1.9 ^a^	10.0 ± 0.9 ^b^
Zinc—Zn	64.1 ± 3.4 ^a^	76.2 ± 9.5 ^a^
Iron—Fe	108.6 ± 8.2 ^b^	198.9 ± 27.1 ^a^
Manganese—Mn	61.5 ± 0.1 ^a^	46.2 ± 4.2 ^b^
Cadmium—Cd	0.1 ± 0.0 ^b^	0.4 ± 0.0 ^a^
Lead—Pb	<1.0	<1.0
Arsenic—As	<1.5	<1.5
Nickel—Ni	<1.5	<1.5
Mercury—Hg	<0.004	0.015 ± 0.00

Note: Different letters in a given row indicate significant differences at *p* < 0.05 (Tukey test).

**Table 3:** Average values (±SD) of water activity (a_w_), moisture content (%, FW), proximal composition and crude fibre (%, DW), and caloric value (kcal) of the insect powders from *A. domesticus* (FI and FII).

Parameters	FI	FII
a_w_	0.4 ± 0.0 ^a^	0.3 ± 0.1 ^a^
Moisture content (%, FW)	5.0 ± 0.2 ^a^	4.3 ± 0.6 ^a^
Protein (%, DW)	57.4 ± 0.6 ^b^	60.8 ± 1.1 ^a^
Lipids (%, DW)	14.1 ± 2.0 ^a^	13.4 ± 0.3 ^a^
Carbohydrates (%, DW)	19.1 ± 1.9 ^a^	16.8 ± 0.6 ^a^
Ash (%, DW)	4.4 ± 0.2 ^b^	4.8 ± 0.1 ^a^
Crude Fibre (%, DW)	14.8 ± 1.7 ^a^	16.4 ± 0.4 ^a^
Caloric value (kcal)	433.2 ± 8.4 ^a^	430.6 ± 4.6 ^a^

Note: Different letters in a given row indicate significant differences at *p* < 0.05 (Tukey test).

**Table 4:** Mean content (±SD) of amino acids (g/100 g DW) and the total sum of essential amino acids (∑EAAs) and non-essential amino acids (∑NEAAs) in *A. domesticus* powders (FI and FII).

Amino Acid (g/100 g DW)	FI	FII
Histidine	2.47 ± 0.47 ^a^	2.33 ± 0.47 ^a^
Threonine	2.37 ± 0.8 ^a^	1.27 ± 0.03 ^a^
Methionine	2.09 ± 0.83 ^a^	1.86 ± 0.22 ^a^
Valine	3.78 ± 0.18 ^a^	2.57 ± 0.64 ^b^
Phenylalanine	2.87 ± 0.5 ^a^	2.68 ± 0.97 ^a^
Isoleucine	3.19 ± 0.48 ^a^	1.76 ± 0.54 ^b^
Leucine	3.68 ± 0.1 ^a^	2.93 ± 0.46 ^a^
Lysine	3.19 ± 0.93 ^a^	2.98 ± 0.55 ^a^
Tryptophan	0.36 ± 0.04 ^a^	0.28 ± 0.04 ^a^
Aspartic acid	1.85 ± 0.75 ^a^	1.97 ± 0.27 ^a^
Glutamic acid	3.38 ± 1.07 ^a^	3.45 ± 0.62 ^a^
Serine	2.87 ± 0.56 ^a^	1.31 ± 0.11 ^b^
Glycine	2.02 ± 0.42 ^a^	1.19 ± 0.16 ^b^
Arginine	4.37 ± 1.43 ^a^	3.32 ± 0.66 ^a^
Alanine	3.45 ± 0.6 ^a^	1.93 ± 0.28 ^b^
Tyrosine	3.49 ± 0.33 ^a^	3.0 ± 0.83 ^a^
∑EAA	24.00 ± 0.81 ^a^	18.70 ± 0.74 ^b^
∑NEAA	21.43 ± 2.52 ^a^	16.17 ± 0.92 ^b^

Note: Different letters in a given row indicate significant differences at *p* < 0.05 (Tukey test).

**Table 5:** Mean content (±SD) of fatty acid (g/100 g of fat) in *A. domesticus* insect powders (FI and FII).

Fatty Acid (g/100 g of Fat)	FI	FII
Myristic acid C14	0.81 ± 0.03 ^b^	1.54 ± 0.29 ^a^
Pentadecanoic acid C15	0.07 ± 0.01 ^b^	0.27 ± 0.07 ^a^
Palmitic acid C16	28.03 ± 0.55 ^a^	25.32 ± 0.58 ^b^
7-Hexadecenoic acid C16:1 (7)	0.39 ± 0.01 ^a^	0.34 ± 0.09 ^a^
11-Hexadecenoic acid C16:1 (11)	0.05 ± 0.01 ^b^	0.21 ± 0.06 ^a^
Palmitoleic acid C16:1 (9)	0.86 ± 0.04 ^b^	2.17 ± 0.34 ^a^
Margaric acid C17	0.27 ± 0.01 ^b^	0.69 ± 0.14 ^a^
Heptadecenoic acid C17:1	0.07 ± 0.03 ^b^	0.37 ± 0.09 ^a^
Stearic acid C18	9.09 ± 0.07 ^a^	7.41 ± 0.04 ^b^
Petroselinic acid C18:1 (6)	0.15 ± 0.07 ^b^	0.37 ± 0.12 ^a^
Oleic acid C18:1 (9)	23.69 ± 0.64 ^a^	24.02 ± 0.65 ^a^
Vaccenic acid C18:1 (11)	0.24 ± 0.13 ^b^	1.34 ± 0.39 ^a^
Trans-Linoleic acid C18:2 (9 t, 12 t)	0.37 ± 0.06 ^a^	0.12 ± 0.04 ^b^
Trans-Linoleic acid C18:2 trans	ND	0.3 ± 0.04
Linoleic acid C18:2 (9, 12)	30.91 ± 0.61 ^a^	26.12 ± 2.58 ^b^
Linolenic acid C18:3 (9, 12, 15)	0.6 ± 0.04 ^b^	1.26 ± 0.06 ^a^
Octadecatetraenoic acid C18:4	ND	0.44 ± 0.07
Arachidic acid C20	0.32 ± 0.01 ^a^	0.29 ± 0.02 ^a^
Gadoleic acid C20:1 (9)	0.09 ± 0.02 ^b^	0.39 ± 0.11 ^a^
Eicosenoic acid C20:1 (11)	0.03 ± 0.02 ^b^	0.68 ± 0.32 ^a^
Behenic acid C20:2	0.1 ± 0.05 ^a^	0.09 ± 0.02 ^a^
Eicosatetraenoic acid C20:4 w3	ND	0.19 ± 0.03
Arachidonic acid C20:4 w6	0.01 ± 0.03 ^b^	0.54 ± 0.09 ^a^
Eicosatrienoic acid (n-3) C20:3 w3	1.0 ± 0.07	ND
Eicosatrienoic acid (n-6) C20:3 (11, 14, 17)	ND	0.55 ± 0.07
Eicosapentaenoic acid C20:5 EPA	ND	1.38 ± 0.21
Erucic acid C22:1	0.34 ± 0.13 ^a^	0.17 ± 0.05 ^a^
Docosapentaenoic acid C22:5	ND	0.14 ± 0.01
Docosahexaenoic acid C22:6 DHA	ND	0.48 ± 0.05
Nervonic acid C24:1	0.32 ± 0.17	ND
Lignoceric acid C24	0.16 ± 0.07 ^a^	0.11 ± 0.09 ^a^

Note: Different letters in a given row indicate significant differences at *p* < 0.05 (Tukey test). Fatty acid levels ≤ 5% are not included in the table. ND corresponds to undetected fatty acid.

**Table 6:** Ratios of SFA, MUFA, and PUFA in *A. domesticus* insect powders (FI and FII) (mean ± SD).

Fatty Acid Types	FI	FII
SFA (%) (saturated fatty acids)	39.1 ± 0.7 ^a^	36.5 ± 1.1 ^b^
MUFA (%) (monounsaturated fatty acids)	26.3 ± 0.5 ^b^	31.3 ± 1.3 ^a^
PUFA (%) (polyunsaturated fatty acids)	33.1 ± 0.7 ^a^	29.6 ± 2.5 ^a^

Note: Different letters in a given row indicate significant differences at *p* < 0.05 (Tukey test).

**Table 7:** Nutritional indices for cricket powders’ (FI and FII) lipids (means ± SD).

Nutritional Indices (Lipids)	FI	FII
IA (index of atherogenicity)	0.53 ± 0.02 ^a^	0.51 ± 0.04 ^a^
IT (index of thrombogenicity)	1.10 ± 0.06 ^a^	0.83 ± 0.03 ^b^
H/H (ratio hypocholesterolemic and hypercholesterolemic fatty acids)	1.93 ± 0.09 ^a^	1.99 ± 0.17 ^a^
ω6/ω3 (ratio omega-6 and omega-3 fatty acids)	16.44 ± 1.98 ^a^	6.80 ± 1.27 ^b^

Note: Different letters in a given row indicate significant differences at *p* < 0.05 (Tukey test).

**Table 8:** Examples of nutritional values for different foods taken from the literature vs. FI and FII.

Index Food Source	IA	IT	H/H	References
Cricket powders FI and FII	0.51–0.53	0.83–1.10	1.93–1.99	data from the present study
*Salmo trutta*	0.64–0.72	0.21–0.30	1.88–2.16	[52]
Heifer (*Limousin heifer*)	0.50–0.57	1.10–1.34	1.27–1.87	[52]
Chicken	0.37–0.39	0.76–0.78	2.66–2.79	[52]
Milk of Jersey cow	2.48–3.44	3.98–4.66	0.41–0.57	[52]
Olive oil	0.16	0.39	6.01	[53]

**Table 9:** Mean results (±SD) of antioxidant activity (AOx) ((DPPH) µmol TE/100 g DW), AOx ((FRAP) mmol FeSO_4_·7H_2_O/100 g DW) and total phenolic content (TPC) (mg GAE/100 g DW) in *A. domesticus* insect powders (FI and FII).

Samples Id	AOx (DPPH) (µmol TE/100 g DW)	AOx (FRAP) (mmol FeSO_4_·7H_2_O/100 g DW)	TPC (mg GAE/100 g DW)
FI	5055.0 ± 2.3 ^a^	72.9 ± 1.9 ^a^	212.0 ± 24.8 ^a^
FII	4928.5 ± 21.7 ^b^	43.0 ± 3.8 ^b^	120.9 ± 5.5 ^b^

Note: Different letters in a given row indicate significant differences at *p* < 0.05 (Tukey test).

**Table 10:** Mean (±SD) mineral content (mg/kg DW) in *A. domesticus* insect powders (FI and FII).

Minerals (mg/kg DW)	FI	FII
Sodium—Na	3819.7 ± 116.0 ^a^	4174.4 ± 230.1 ^a^
Potassium—K	10,254.9 ± 478.1 ^b^	11,452.8 ± 443.0 ^a^
Calcium—Ca	2193.4 ± 117.8 ^a^	2382.5 ± 55.9 ^a^
Magnesium—Mg	1167.4 ± 15.6 ^b^	1316.7 ± 36.3 ^a^
Phosphorus—P	9956.6 ± 223.5 ^b^	10,965.5 ± 233.8 ^a^
Sulfur—S	5482.7 ± 127.0 ^b^	6098.4 ± 208.6 ^a^
Copper—Cu	26.7 ± 1.4 ^a^	27.0 ± 1.5 ^a^
Zinc—Zn	196.0 ± 5.2 ^a^	187.5 ± 10.2 ^a^
Iron—Fe	69.0 ± 9.7 ^a^	53.2 ± 10.9 ^a^
Manganese—Mn	27.7 ± 3.5 ^a^	16.6 ± 7.3 ^a^
Chromium—Cr	3.12 ± 0.1 ^a^	3.00 ± 0.1 ^b^
Cadmium—Cd	<1.0	<1.0
Lead—Pb	<1.0	<1.0
Arsenic—As	<1.5	<1.5
Nickel—Ni	<1.5	<1.5
Mercury—Hg	0.021 ± 0.01 ^b^	0.228 ± 0.07 ^a^

Note: Different letters in a given row indicate significant differences at *p* < 0.05 (Tukey test).

**Table 11:** Mean (±SD) of microbiological assessments in *A. domesticus* insect powders (FI and FII).

Microbiological Parameter (log10 CFU/g)	FI	FII
Microorganisms at 30 °C	3.6 ± 0.4 ^a^	3.2 ± 0.3 ^a^
Molds and Yeasts	1.0 ± 1.0 ^a^	0.3 ± 0.6 ^a^

Note: Different letters in a given row indicate significant differences at *p* < 0.05 (Tukey test).

## Data Availability

The original contributions presented in this study are included in the article/Supplementary Materials. Further inquiries can be directed to the corresponding author.

## Supplementary Materials

The following supporting information can be downloaded at: https://www.mdpi.com/article/10.3390/foods14071242/s1, Table S1: Coefficients of the variables in the principal component analysis (PCA) (12 samples and 13 variables).
