# Supplementary material for: Nutritional and Microbial Quality of Edible Insect Powder from Plant-Based Industrial By-Product and Fish Biowaste Diets
Source: Foods. 2025 Apr 2;14(7):1242. doi: 10.3390/foods14071242 (PMC11988334; doi:10.3390/foods14071242)
Supplement: Supplementary file 1 [file foods-14-01242-s001.zip › foods-3549618-supplementary.pdf]

foods

Article

Nutritional and Microbial Quality of Edible Insect Powder from Plant-Based Industrial By-Product and Fish Biowaste Diets

Rafaela Andrade [1,2], Luisa Louro Martins [2], Miguel Pedro Mourato [2], Helena Lourenço [3], Ana Cristina Ramos [1,4], Cristina Roseiro [1,4], Nelson Pereira [1,2], Gonçalo J. Costa [5], Raphael Lucas [5], Nuno Alvarenga [1,4], João Reis [6], Ana Neves [6], Margarida Oliveira [2,6,7], Igor Dias [6,7,8,9] and Marta Abreu [1,2,*]

[1] Instituto Nacional de Investigação Agrária e Veterinária, Unidade de Tecnologia e Inovação, 2780-157 Oeiras, Portugal; andsrafaela@gmail.com (R.A.); cristina.ramos@iniav.pt (A.C.R.); cristina.roseiro@iniav.pt (C.R.); isa128286@isa.ulisboa.pt (N.P.); nuno.alvarenga@iniav.pt (N.A.)
[2] LEAF—Linking Landscape, Environment, Agriculture and Food Research Center, Associated Laboratory TERRA, Instituto Superior de Agronomia, ULisboa, 1349-017 Lisboa, Portugal; luisalouro@isa.ulisboa.pt (L.L.M.); mmourato@isa.ulisboa.pt (M.P.M.); margarida.oliveira@esa.ipsantarem.pt.com (M.O.)
[3] Instituto Português do Mar e da Atmosfera, I. P. (IPMA, I. P.), Avenida Alfredo Magalhães Ramalho 6, 1495-165 Algés, Portugal; helena@ipma.pt
[4] GeoBioTec—Geobiociências, Geoengenharias e Geotecnologias, NOVA School of Science and Technology, Universidade Nova de Lisboa, 2829-516 Caparica, Portugal
[5] The Cricket Farming Co., Quinta do Galinheiro, S. Pedro, 1001-904 Santarém, Portugal; goncalocosta@thecricketfarmingco.pt (G.J.C.); raphaellucas@thecricketfarmingco.pt (R.L.)
[6] Escola Superior Agrária de Santarém, UI_IPS—Instituto Politécnico de Santarém, Quinta do Galinheiro, S. Pedro, 1001-904 Santarém, Portugal; joao.reis@esa.ipsantarem.pt (J.R.); ana.neves@esa.ipsantarem.pt (A.N.); igor.dias@esa.ipsantarem.pt (I.D.)
[7] Centro de Estudos de Recursos Naturais Ambiente e Sociedade (CERNAS), Instituto Politécnico de Santarém, Quinta do Galinheiro, S. Pedro, 1001-904 Santarém, Portugal
[8] CIEQV—Life Quality Research Centre, Avenida Dr. Mário Soares n 110, 2040-413 Rio Maior, Portugal
[9] MED–Instituto Mediterrâneo para a Agricultura, Ambiente e Desenvolvimento & CHANGE–Global Change & Sustainability Institute, Universidade de Évora, Pólo da Mitra, Apartado 94, 7006-554 Évora, Portugal
* Correspondence: marta.abreu@iniav.pt

Supplementary Material

Tables

Table S1. Coefficients of the variables in the Principal Component Analysis (PCA) (12 samples and 13 variables).

Attributes	PC1	PC2
Humidity	-0.83*	-0.55
Protein	0.68	0.73*
Lipids	-0.74*	0.66
Carbohydrates	-0.34	-0.94*
Fibre	-0.96*	0.24
Ash	-0.99*	-0.04
Caloric Value	-0.53	0.83*
Na (Sodium)	-0.62	0.78*
K (Potassium)	-0.75*	-0.40
Ca (Calcium)	-0.97*	-0.20
Mg (Magnesium)	-0.95*	-0.28
P (Phosphorus)	-0.93*	0.37
S (Sulfur)	-0.10	0.98*

* marked loading are > 0.70.
